# Secure Image Encryption Using Chaotic, Hybrid Chaotic and Block Cipher Approach

**DOI:** 10.3390/jimaging8060167

**Published:** 2022-06-10

**Authors:** Nirmal Chaudhary, Tej Bahadur Shahi, Arjun Neupane

**Affiliations:** 1 Central Department of Computer Science and IT, Tribhuvan University, Kathmandu 44600, Nepal; nirmalchaudhary893@gmail.com; 2 School of Engineering and Technology, Central Queensland University, Rockhampton, QLD 4701, Australia; a.neupane@cqu.edu.au

**Keywords:** image encryption, chaos theory, block cipher, Arnold cat map, logistic map, AES

## Abstract

Secure image transmission is one of the most challenging problems in the age of communication technology. Millions of people use and transfer images for either personal or commercial purposes over the internet. One way of achieving secure image transmission over the network is encryption techniques that convert the original image into a non-understandable or scrambled form, called a cipher image, so that even if the attacker gets access to the cipher they would not be able to retrieve the original image. In this study, chaos-based image encryption and block cipher techniques are implemented and analyzed for image encryption. Arnold cat map in combination with a logistic map are used as native chaotic and hybrid chaotic approaches respectively whereas advanced encryption standard (AES) is used as a block cipher approach. The chaotic and AES methods are applied to encrypt images and are subjected to measures of different performance parameters such as peak signal to noise ratio (PSNR), number of pixels change rate (NPCR), unified average changing intensity (UACI), and histogram and computation time analysis to measure the strength of each algorithm. The results show that the hybrid chaotic map has better NPCR and UACI values which makes it more robust to differential attacks or chosen plain text attacks. The Arnold cat map is computationally efficient in comparison to the other two approaches. However, AES has a lower PSNR value (7.53 to 11.93) and has more variation between histograms of original and cipher images, thereby indicating that it is more resistant to statistical attacks than the other two approaches.

## 1. Introduction

Digital image security has become a critical problem because many digital services, such as multimedia systems [1] and medical [2,3] and public internet communication [4], require reliable security for the storage and transmission of digital images. Moreover, the growth of the internet and mobile communications demands a secure communication system. Such a system needs to ensure that end user privacy has not been compromised in any way [5]. To this end, providing a higher level of security and maintaining the quality of data without losing the original image’s parametric properties are equally important. Therefore, it is quite essential to build a secure image encryption framework with better efficiency, confidentiality and quality [6]. The low scale performance and vulnerability of existing cryptosystems to security threats make them ineffective for image encryption [7]. Herein, the chaotic system with complex properties of ergodicity, unpredictability and sensitivity has opened the potential for their use in cryptography [8]. The standard encryption algorithms have mainly relied on two important properties of confusion and diffusion for their encryption capability [9], which are also included in the chaotic system, such as being ergodic and extremely sensitive to initial conditions and the system parameters to provide a strong secret key. Various chaos based image encryption systems have been proposed [8,10,11,12,13,14,15,16]. In these works, the chaotic map, an evolution function that demonstrates some of the chaotic behavior either in discrete-time or a continuous-time domain, was used for image encryption. Different chaotic maps such as 1D, 2D and 3D were experimented with for image encryption. Besides these single chaotic maps, such as the tent map [17], logistic map [18], Arnold cat map [8] and Henon map [19], there are few works that use a hybrid chaotic map by intermixing the result of one chaotic map with another chaotic map [20,21]. The hybrid chaotic map seems to have better security measurement parameters than the standalone chaos-based image encryption system.

A chaos-based image encryption algorithm was proposed in [8], where the image pixel was shuffled with a chaotic Arnold cat map and then it was further processed with Chen’s chaotic system [22]. Their result shows that the combination of one chaotic map with another enhances the security of the image encryption method. A logistic map-based secure image encryption was proposed in [18]. Here, the authors used two chaotic maps with an external secret key. Furthermore, they employed eight different operations to encrypt the pixels of the image decided by the outcome of the logistic map.

A combination of the chaotic map with an advanced encryption system (AES) was investigated by Arab et al. [12] for image encryption. They generated a block cipher encryption key with an Arnold cat map and performed the image encryption with a modified AES algorithm. In modified AES, in each round of encryption, the round keys were generated with a chaos system, making their algorithm resistant to differential attacks. A chaotic map-based light weight model for image encryption was implemented in [23]. They performed both substitution and permutation of the pixel values in one scan of the image with the aid of a chaotic map, thereby reducing the computation cost.

With the existing works, it can be seen that a chaos-based system for image encryption has gained popularity in the recent past. Since a chaotic map uses the chaotic function of different chaotic methods, the behavior of the chaotic function can be reflected with the help of chaotic encryption which uses a mathematical function of the chaotic map to shuffle and scramble the image thereby changing the pixel value and position to make encryption better, so that an attacker cannot read the original pixel information in the image. Furthermore, chaotic encryption uses a number of parameters as a secret key which ensures high randomization of the image data during encryption and decryption [24]. Hence, the chaotic-based cryptosystem adds a new paradigm for image encryption. However, we observe the following limitation while summarising the existing works in chaos-based image encryption. First, most existing works [13,14] use a single chaotic map for image encryption which needs further security assessment to provide sufficient confidence in the encryption process. Second, existing works either combined the chaotic map with a block cipher technique [12] or another chaotic map [20,21,25] as a hybrid chaotic map for image encryption. Therefore, there is a need for further analysis to measure the encryption efficiency of each of them individually.

We aim to deal with the aforementioned limitation with the following objectives. We first propose to use a chaotic map (Arnold cat map), its combination with a logistic map (hybrid chaotic map) and an advanced encryption standard (AES) block cipher encryption method for image encryption. Second, we compare these three encryption algorithms for benchmark image datasets to evaluate their efficiency. Third, we evaluate their efficiency using four widely used parameters: visual assessment, differential analysis, computation speed, and statistical analysis.

The main contributions of our work are summarized as follows:

(i) We propose to use three different encryption methods—Arnold cat map, hybrid chaotic map, and Advanced encryption standard (AES)—for image encryption;
(ii) We evaluate the performance of each encryption method on four widely-used image datasets using four evaluation metrics such as visual assessment, differential analysis, computational speed, and statistical analysis.

The rest of the paper is organized as follows. Section 2 discusses the existing literature on chaos system based image encryption. Methods such as block cipher, chaotic map and hybrid chaotic maps for image encryption are discussed in Section 3. The analysis and comparison between three methods for image encryption are presented in Section 4. The results and discussion are presented in Section 5. Finally, Section 6 concludes our work and presents further recommendations.

## 2. Related Work

Image encryption with a block cipher and a chaotic map was proposed in [26] where chaotic maps were used as a source of entropy thereby reducing the number of rounds in block cipher encryption. However, their methods need to be further analysed for crypt-analytic attacks. The authors of [7] implemented an efficient permutation at intra-inter bit-level based confusion strategy for chaos-based image encryption. They employed a random number generating strategy in the encryption process’s starting stages, aiming to reduce the number of iterations in Fridrich’s structure. However, Chen et al. [15] proposed to use the affine transform and the gyrator transform for color image encryption. The affine transform was used twice in the encryption process. The parameters of these transforms served as the secret key. Initially, the RGB image was broken down into its three independent components (R,G,B) and converted into the real and imaginary parts of a complex function with affine transform. Finally, the gyrator transform was applied twice to enhance the security of the encryption process. Ansari et al. [11] proposed a distinct approach for encrypting an image that uses chaotic maps in the Frequency Domain. The Discrete Cosine Transform (DCT) of the image is evaluated and the shuffling of the image is performed by a 2D baker’s map. Two baker’s maps are used where the first uses the primary set of keys and the other is used with a Gaussian image generated with mean-variance. The gain of both baker’s maps and DCT are XORed repetitively. The scattering pattern is formed by a number generator which engenders a random pattern based on Gaussian distribution. The suggested encryption method uses two baker’s maps thus capable of accommodating the key-space up to 128 bits. Tang et al. [27] proposed an encryption method for four greyscale images for creating a more secure image transmission. The infant input gray-scale image is prorated into bit-planes and the swapping of bit-blocks among various bit-planes takes place randomly. An XOR logical operation is enforced between these scrambled data and a matrix controlled using a chaotic map which operates as a secret key. The components of four gray-scale images, i.e., green, red, alpha and blue, are used to generate an encrypted image. Zhu et al. [28] have suggested a unique method established on the chaos technique. This method uses the principle of a puzzle cube for shuffling all pixel values in a 3-dimensional (3D) plane and modifying them using the pseudo-random pattern obtained by a compound chaotic map, i.e., a combination of sine map, chaotic map and cosine map. The method generally achieves the goal of high speed and various experimental analysis prove that it achieves a very high-security level. The authors of [12] proposed an image encryption algorithm based on the modified AES algorithm with a chaotic map. They generated an encryption key using the Arnold chaos sequence and then the AES algorithm is used for image encryption in which the round keys produced by the chaos system are used. They showed that it is highly resistant to differential attack along with lower time complexity.

## 3. Methods

**Figure 1:**
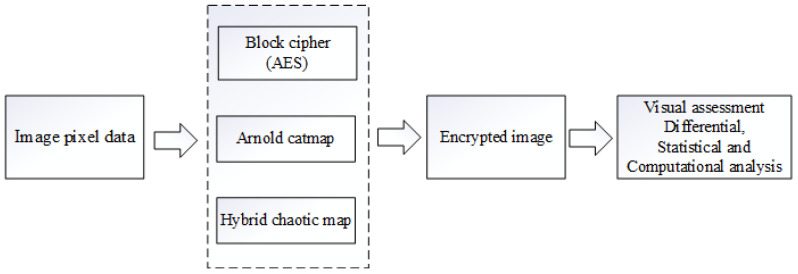
The high level block diagram of the proposed method.

In this section, we discuss the three image encryption methods. The overall methodology of the proposed work is depicted in Figure 1.

### 3.1. Block Cipher Image Encryption Using AES

The advanced encryption standard (AES) [29] is a symmetric block cipher based on substitution and permutation network. It encrypts the images with a number of rounds where each round processes the images by dividing them into blocks of size 128 bits with three different key size lengths 128, 192 or 256. The number of execution rounds is based on key size length. The number of execution rounds of the AES algorithm is 10, 12 or 14 for a key size of 128, 192 and 256 respectively. In this work, we use the AES with a block size of 128 bits and a key size of 128 bits. Since the larger key size and more execution rounds demand more computational time, we choose the basic version of AES for a fair comparison with other image encryption methods in this work.

An individual round of AES image encryption consists of four stages in sequence: Substitute Bytes, Shift Row, Mix Columns and AddRoundKey. The substitute byte transformation consists of non-linear byte substitution, which operated on each of the state bytes independently. The substitution table, called s-box, is used to achieve these transformations. The S-box table contains 256 numbers (from 0 to 255) and their corresponding resulting values. Next, Shift Rows transformation is operated on the rows of the state cyclically starting from the left. The first row (Row-0) is left unchanged while each byte of the second row (Row-1) does a shift of one byte to the left. Similarly, Row-2 does a shift of two bytes to the left and Row-3 does a shift of three bytes to the left.

In the Mix-Columns transformation, the columns of the state are considered as polynomials over the Galois field ($2^{8}$) and are multiplied by (modulo + 1) with a fixed polynomial *c*(*x*), as defined in Equation (1):

(1) c(x)=03+01+01x+02.

**Figure 2:**
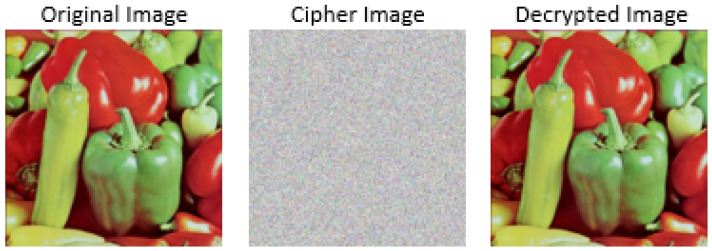
Illustration of encryption and decryption with AES for pepper image.

In the AddRoundKey transformation, a round key or sub-key is combined with the state resulting from the previous step, mix-column transformation using a simple bit-wise XOR operation. The key expansion algorithm is used to derive a round key for each round of the AES algorithm. The decryption process in AES involves the inverse application of the above four stages. An illustration of the encryption and decryption of images with AES is shown in Figure 2.

### 3.2. Chaotic Map Encryption Using Arnold Cat Map

It was first proposed by Vladimir Arnold and implemented on a cat image [30], hence it is named the Arnold cat map. It is a mathematical transformation that can be applied to image pixels [31]. When the Arnold cat map transformation is applied to image pixels, they appear to be randomly rearranged. However, if this transformation is repeated enough times, the original image can be restored. Moreover, a discrete Arnold cat map stretches and folds the trajectories in phase space where a square represents the phase space for this simple discrete system and stretching and folding reflect the scrambling effect of the Arnold cat map. The Arnold cat map takes concepts from linear algebra and uses them to change the positions of the pixel values of the original image. After applying the Arnold cat map, the result will be a shuffled image that contains all of the same pixel values of the original image. The transformation that is used by the Arnold cat map is based on a matrix with a determinant of Equation (2) that makes this transformation reversible and can be described as:

(2) $$\left(\begin{array}{c} x^{\prime }\\ y^{\prime } \end{array}\right)=\left(\begin{array}{cc} 1 & p\\ Q & PQ+1 \end{array}\right)\left(\begin{array}{c} x\\ y \end{array}\right)mod\left(n\right).$$

Here, *P* and *Q* are integers and (*x*, *y*) is the original position that is mapped to the new position (x′, y′). *P* and *Q* represent the parameter used in the transformation and are generally taken as prime numbers.

The image pixel position is the input to the cat map equation; the cat map takes the linear sequences of the image pixel position and shuffles the position, resulting in the encrypted image. The process is iterated until the last pixel position. The decryption process is reversed of the encryption process as defined in Equation (3).

(3) $$\left(\begin{array}{c} x\\ y \end{array}\right)=\left(\begin{array}{cc} PQ+1 & -p\\ -Q & 1 \end{array}\right)\left(\begin{array}{c} x^{\prime }\\ y^{\prime } \end{array}\right)mod\left(n\right).$$

**Figure 3:**
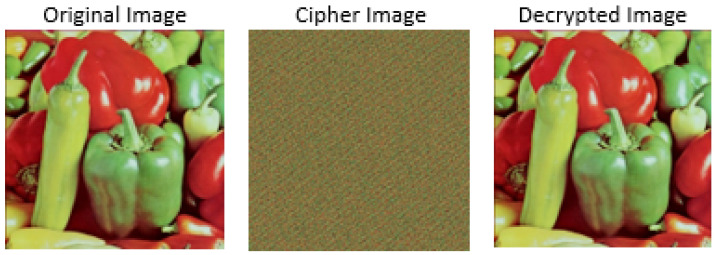
Illustration of encryption and decryption with Arnold cat map for pepper image.

The summary of the image encryption process with the Arnold cat map is depicted in Algorithm 1. Similarly, an illustration of the encryption and decryption of the pepper image with the Arnold cat map is shown in Figure 3.    
 **Algorithm 1:** ACM(I) **Input**: **Input:** $I_{p}\leftarrow$ Any Square image **Output**: **Output:** $I_{e}\leftarrow$ New encrypted image1  num← n, row← r, col← c2  **for** i=0 to num **do**3     **for** j=0 to row **do**4        **for** k=0 to col **do**    Shuffle the positions of the pixels of the image using Equation (2)5        **end for**6     **end for**7  **end for**8  **Return** $I_{e}$

### 3.3. Hybrid Chaotic Map Encryption

The Arnold cat map takes concepts from linear algebra and uses them to change the positions of the pixel values of the original image. It shows certain chaotic behaviours such as sensitivity to initial conditions, periodicity and transitivity. The Arnold cat map encryption algorithm has periodicity, which reduces its encryption security. The logistic map can be used to enhance the security of the chaos system over the Arnold cat map transformation. The logistic map [18] is a one-dimensional discrete map that uses polynomial mapping of degree 2, often referred to as an archetypal example of how complex chaotic behavior can arise from very simple non-linear dynamic equations.

Let us consider an N×N image and *x* and *y* represent the row and column number of the pixels in the image respectively. Thus, *x* and *y* both range from 1 to *N*. Then, mathematically, the logistic map can be defined as in Equation (4):

(4) x(n+1)=λx(n)(1−x(n)),n=0,1,2,3⋯,

where *x* is the map input variable and *x*(0) acts as its initial condition, λ is system parameter ∈(0<λ<4 ) and ’*n*’ is number of iterations needs to be applied. The logistic map shows the road to chaos depending upon the value of λ. When λ is <3, the value of *x* reaches fixed points after several iterations and emerges in a stable period of cycle-2, without showing any chaotic behaviours. If we keep increasing the value of λ, the map exhibits chaotic dynamics for 3.57 < λ < 4 and x(n)∈(0,1) for all *n*.

While using the logistic map for image encryption, the initial value λ and *x*(0) represent the secret key. Therefore, the exact values of these two parameters, λ and *x*(0), are needed at the receiver’s end to successfully decrypt the message. This makes the encryption algorithm entirely key dependent thereby making it very difficult to extract the information from the encrypted images by the attacker or middle-man.

**Figure 4:**
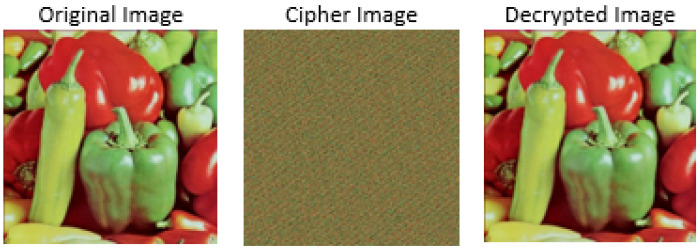
Illustration of encryption and decryption with hybrid chaotic method for pepper image.

The summary of image encryption with the hybrid chaotic map is depicted in the Algorithm 2. Similarly, an illustration of the encryption and decryption of the pepper image with a hybrid chaotic map is shown in Figure 4.
 **Algorithm 2:** Hybrid chaotic map (I)  **Input**: **Input:** $I_{p}\leftarrow$ Any Square image  **Output**: **Output:** $I_{e}\leftarrow$ New encrypted image  1 num← n, row← r, col← c  2 $I_{a}\leftarrow$ ACM(I)  3 $I_{1}\left(m,m\right)\leftarrow$ Shuffle(I)  4 $(C_{1},C_{2})\leftarrow$ LogisticMap()  5 $\left(S_{x},S_{y}\right)\leftarrow \left(Sort\left(C_{1}\right),Sort\left(C_{2}\right)\right)$  6 **for** i=2 to *m* **do**  7   **for** j=0 to *m* **do**  8     **if** (mod(j,2)==0) **then**  9      $I^{\prime \prime }\left(i,j\right)=I^{\prime }\left(i-1,j-1\right)\left(XOR\right)i_{x}\left(k\right),k=1,2,3\ldots \ldots ..\left(m/2\right);$10    **else**11     $I^{\prime \prime }\left(i,j\right)=I^{\prime }\left(i-1,j-1\right)\left(XOR\right)i_{y}\left(k\right),k=1,2,3\ldots \ldots ..\left(m/2\right);$12    **end if**13  **end for**14 **end for**15 **return** $I_{e}$

## 4. Security Measures and Analysis

Test images were taken from a widely used image repository called the “SIPI image database” [32], and the “DICOM (Digital Imaging and Communications in Medicine) image database” [33], because these are the standard image chosen by most of the existing works [21,31] to evaluate the strength of image encryption algorithms. Besides this, we also tested the encryption algorithms on user-captured images to further validate the results. The images taken were of different sizes and dimensions as summarized in Table 1.

The algorithms implemented during this study were analysed in various dimensions and sizes. Computational analysis based on encryption and decryption time, differential analysis based on Number of Pixels Change Rate (NPCR) and Unified Average Changing Intensity (UACI), statistical analysis based on histogram analysis and visual assessment analysis based on Peak Signal to Noise Ratio (PSNR) of input and enciphered images were conducted.

### 4.1. Visual Assessment Analysis

Visual assessment analysis is the ratio of the mean square difference of the component for the two images to the maximum mean square difference that can exist between any two images. When comparing the PSNR value derived from the original image and the cipher image, the lower PSNR value denotes the greater difference between them which ultimately implies a more secure image encryption. The MSE and PSNR are defined in Equations (5) and (6) respectively.

(5) $$MSE=\frac{1}{W\times H}\sum_{i=1}^{W}\sum_{j=1}^{H}{\left(I\left(i,j\right)-I^{\prime }\left(i,j\right)\right)}^{2}$$

(6) $$PSNR=10\times \log\frac{\left(255^{2}\right)}{MSE},$$

where *I* and *I*${}^{\prime }$ represent the original image and cipher image and *W* and *H* represent the width and height of the image, respectively.

The visual assessment analysis is carried out by measuring the PSNR value. The PSNR measures show that the AES has better results than both the Arnold cat map and the hybrid chaotic map, which indicates that the noise ratio after encryption is less in AES and it results in a better decrypted image than the other two encryption methods (Ref to Table 2).

### 4.2. Differential Analysis

Differential analysis is a technique that observes how differences in input affect the differences in the output. NPCR (Number of pixel changes) and UACI (Unified Average Change Intensity) are the two widely used security measures in the image encryption community for differential analysis. NPCR concentrates on the absolute number of pixels which changes the value in differential attacks while the UACI focuses on the averaged difference between two paired cipher images. NPCR and UACI can be calculated using Equations (7) and (9).

(7) $$NPCR=\frac{\sum_{i,j}D\left(i,j\right)}{W\times H}\times 100\%$$

(8) $$D\left(i,j\right)=\left\{\begin{array}{cc} 0, & \mathrm{if}\;C_{1}\left(i,j\right)=C_{2}\left(i,j\right)\\ 1, & \mathrm{if}\;C_{1}\left(i,j\right)\neq C_{2}\left(i,j\right) \end{array}\right.$$

(9) $$UACI=\frac{1}{W\times H}\left(\sum_{i,j}\frac{|C_{1}\left(i,j\right)-C_{2}\left(i,j\right)|}{255}\right)\times 100\%,$$

where $C_{1}$ and $C_{2}$ represent the cipher images before and after changing the one pixel value and W and H represent the width and height of the image respectively.

While looking at Table 3, it is found that the hybrid chaotic map poses the highest NPCR value for all four images, followed by the Arnold cat map. The AES has the lowest NPCR value. Here, the higher NPCR value represents that the encryption algorithm has a higher resistance for defending against the differential attack of the intruders. Comparing the UACI values of the three algorithms, the hybrid chaotic map has higher UACI values than the other two algorithms (Arnold cat map and AES) for all images except the “Baboon.jpg” image.

### 4.3. Computational Speed Analysis

Computation analysis is the measure of time consumed by the algorithms. We measure the value of encryption and decryption time for the computational performance for a different set of images in milliseconds to find which technique is computationally efficient. Referring to the results in Table 4, it can be observed that the AES block cipher algorithm has a relatively higher computational time than that of the Arnold cat map and hybrid chaotic algorithms. For instance, AES has 2485 milliseconds of encryption time and 2037 milliseconds of decryption time for “Boobon.jpg” images while the Arnold cat map took only 2235 milliseconds and 1580 milliseconds for the encryption and decryption of the “Boobon.jpg” image respectively. The overall results on computational time for all images show that the native Arnold cat map has a lower computational cost compared to the other two algorithms: AES and hybrid chaotic cat map (Ref to Table 4).

### 4.4. Statistical Analysis

**Figure 5:**
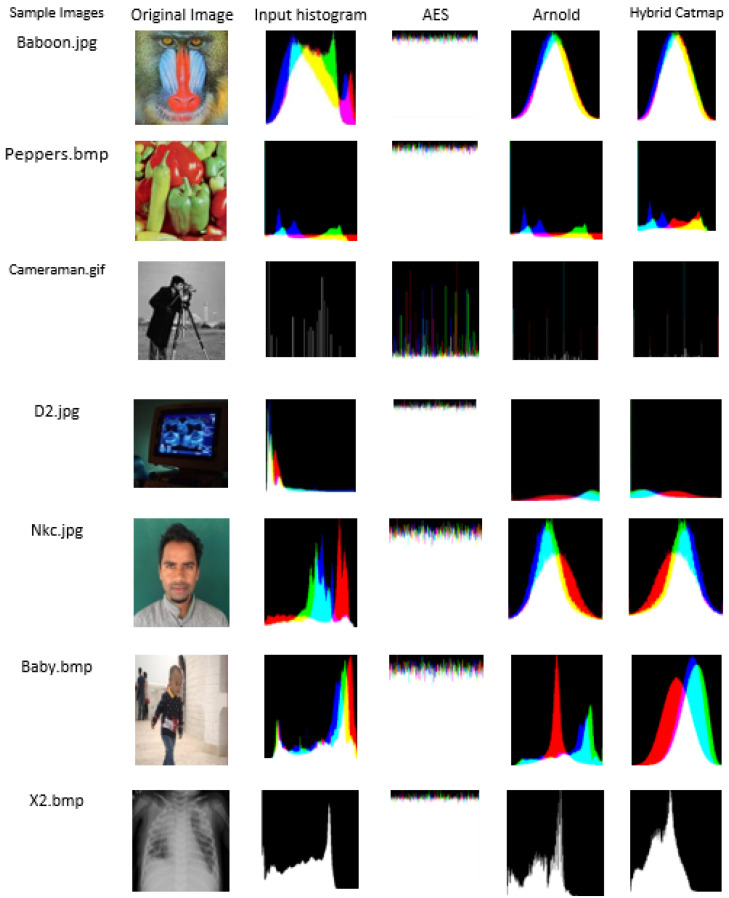
Histogram visualization of samples image using three encryption methods.

Statistical analysis was carried out with the help of histogram analysis. It is a graphical representation of the distribution of the value of pixel information. It must be perfectly uniform for the image to be exact to the original image. AES provides better statistical analysis than the Arnold cat map and the hybrid chaotic cat map. The histogram of the original images and encrypted images with AES provides no relation with each other and hence is able to confuse the intruders from finding any clue to attack it. Chaotic and hybrid chaotic resemble similar histograms for original and encrypted images (Ref to Figure 5).

## 5. Result and Discussion

After performing the different analyses with three encryption algorithms, the result shows that each algorithm has its own capabilities and functionality for protecting the image properties. The result shows that AES has maintained a good image quality after decrypting the image compared to the chaotic cat map and hybrid chaotic map as this is supported by the lower PSNR value for AES. The PSNR value of AES is in the range of 7.21 (for D2.jpg) to 11.93 (for Baboon.jpg), representing a good value which confirms that the original image and decrypted image are similar without losing much of their image properties. However, the chaotic map and the hybrid chaotic map have a significantly higher PSNR value which ranges from 10 upwards; this value of PSNR results in a change to the visual properties of the image.

The hybrid chaotic map has a greater value of NPCR and UACI in the range of 95–99 and 28–34, respectively, for a different set of the images, which is a significantly greater value compared to the chaotic map and AES. This result shows that the hybrid chaotic map stands well in the differential analysis compared to the other two approaches in protecting the image from intruders by creating more chaos in the image pixel properties. It is also noticed that some of the values for NPCR and UACI for the image sets are significantly lower than the standard range of values, which determined the failure of the differential analysis test for that image (Ref. to Table 3).

The Arnold cat map proved to be a computationally efficient algorithm in terms of encryption and decryption. The native chaotic approach saves computational time. The analysis was performed in terms of milliseconds and the result shows that the chaotic map has the lowest value compared to the other two methods.

AES resembles quality performance in the histogram analysis of the image as the histograms of the cipher and original image show more variation which ultimately lessens the chances of providing a clue to the intruders and prevents them from attacking. The chaotic map and hybrid chaotic map had similar histograms for encrypted and decrypted images compared to AES, which shows that there are fewer variations and more chances to predict the image properties by intruders.

In summary, it can be seen from the above analysis that the hybrid chaotic map is better at resisting differential attacks. The chaotic cat map preserves the encryption and decryption time which makes it computationally efficient compared to the other two methods. AES has a good characteristic of preserving image properties after decryption as it has a lower PSNR value and significant differences between the histogram of the cipher and the original image.

## 6. Conclusions and Future Work

In this paper, we proposed three approaches: chaotic, hybrid chaotic and block cipher for secure image encryption. The algorithms were implemented and analyzed with various parameters, such as peak signal to noise ratio (PSNR), number of pixels change rate (NPCR), unified average changing intensity (UACI), and histogram and computation time analysis to measure their strength in terms of image encryption. The results showed that AES is computationally inefficient but can preserve the image properties after encryption as it has a lower PSNR value and high histogram differences. The hybrid chaotic method has high NPCR and UACI values which show its strength in resisting differential attacks, although it seems to preserve fewer image properties. The Arnold cat map method is computationally efficient compared to the other two approaches. Overall analysis and results from the above discussion conclude that the chaotic, hybrid chaotic, and block cipher approaches positively impact image encryption.

To maintain certain secured properties of image data with chaotic, hybrid chaotic and block cipher approaches, a few works can be carried out in the future. The approaches of higher dimensional 3D and 4D chaotic maps can be considered to build a secure and efficient image encryption framework as the next level of work. The chaos-based cryptosystem also equally creates a significant impact on the image encryption process so if it should also be carried out, it could make a remarkable impact on this research. The mechanism to preserve the loss of image properties that are likely to appear after decrypting the image is also a major issue to be addressed by the chaotic and hybrid chaotic methods. Additionally, the cryptanalysis of the analyzed algorithms can also be performed in the future to determine security threats and attack strengths.

## Figures and Tables

**Table 1:** The brief description of sample images used in this work.

Image Name	Dimension	Database
Baboon. jpg	512×512	SIPI
peppers.bmp	512×512	SIPI
cameramen.gif	256×256	SIPI
D2.jpg	800×800	DICOM
Nkc.jpg	300×300	User-captured
Baby.bmp	2000×2000	User-captured
X2.bmp	1000×1000	Chest X-ray image

**Table 2:** PSNR measures of seven sample images using three encryption methods.

Sample Images	AES	Arnold Cat Map	Hybrid Chaotic Map
Baboon.jpg	11.93	12.50	13.08
peppers.bmp	10.82	11.09	11.89
Cameraman.gif	9.52	10.00	10.56
D2.jpg	7.21	12.28	12.49
Nkc.jpg	11.32	12.53	12.75
Baby.bmp	7.89	9.71	9.99
X2.bmp	7.53	10.80	11.08

**Table 3:** NPCR and UACI measures of seven sample images using three encryption methods.

Samplle Images	AES		Arnold Cat Map		Hybrid Chaotic Map	
	NPCR	UACI	NPCR	UACI	NPCR	UACI
Baboon.jpg	86.68	64.14	96.99	22.98	99.98	26.67
peppers.bmp	0.0030	0.0020	84.80	25.96	99.98	34.35
Cameraman.gif	0.0063	0.0021	0.0089	0.0030	0.67	0.334
D2.jpg	93.32	0.067	98.95	0.234	99.99	0.343
Nkc.jpg	83.23	20.76	90.00	30.32	99.95	32.85
Baby.jpg	80.21	21.34	92.14	29.83	99.34	32.44
X2.bmp	$6.89\times 10^{-7}$	$2.43\times 10^{-7}$	$9.99\times 10^{-5}$	$1.4\times 10^{-7}$	$5.04\times 10^{-4}$	$2.10\times 10^{-4}$

**Table 4:** Encryption and Decryption time measures of seven sample images using three encryption methods. Note that Encrypt. and Decrypt. denotes the encryption and decryption time (in milliseconds) taken by each algorithm, respectively.

Samplle Images	AES		Arnold Cat Map		Hybrid Chaotic Map	
	Encrypt.	Decrypt.	Encrypt.	Decrypt.	Encrypt.	Decrypt.
Baboon.jpg	2485	2037	2235	1580	2456	1860
peppers.bmp	1789	1560	1570	1324	1756	1532
Cameraman.gif	1434	1221	1257	908	1321	1009
D2.jpg	8829	5229	4853	3595	5334	4563
Nkc.jpg	6436	5720	3754	3066	3953	3609
Baby.bmp	69,007	12,990	10,230	5907	34,390	8439
X2.bmp	7248	4843	6385	3980	6951	5745
